# Effectiveness of tranexamic acid on chronic subdural hematoma recurrence: a meta-analysis and systematic review

**DOI:** 10.3389/fneur.2024.1359354

**Published:** 2024-04-22

**Authors:** Wani Pan, Jinyang Hu, Xin Huang, Erlang Jin, Longfei Yao, Jing Han, Tiantian Liu

**Affiliations:** ^1^Department of Neurosurgery, The First College of Clinical Medical Science, Three Gorges University, Yichang Central People's Hospital, Yichang, China; ^2^Department of Oncology, The First College of Clinical Medical Science, China Three Gorges University, Yichang Central People's Hospital, Yichang, China

**Keywords:** tranexamic acid, chronic subdural hematoma, recurrence, meta-analysis, systematic review

## Abstract

**Objectives:**

Our objective was to compare the effectiveness of TXA in improving recurrence in patients with chronic subdural hematoma (CSDH).

**Methods:**

Eligible randomized controlled trials (RCTs), prospective trials and retrospective cohort studies were searched in PubMed, Cochrane Library, Embase, and CNKI from database inception to December 2023. After the available studies following inclusion and exclusion criteria were screened, the main outcome measures were strictly extracted. Reman v5.4. was used to assess the overall recurrence rate. A random-effects model was used to assess pooled ORs, with the Mantel–Haenszel estimation method applied. Cochran Q (Chi-square) test and I2 statistics were used to assess inter-study heterogeneity. Funnel plots were used to evaluate publication bias.

**Results:**

From the 141 articles found during initial citation screening, 9 literatures were ultimately included in our study. Our NMA results illustrated that patients with newly diagnosed Chronic subdural hematoma revealed a significantly improved recurrence rate when patients were treated with Tranexamic acid (OR: 0.33; 95% CI 0.26–0.41; *p* < 0.00001) compared with standard neurosurgical treatment. There was no significant difference in the incidence rates of thrombosis (OR: 0.84; 95% CI 0.63–1.12; *p* = 0.23) and mortality (OR: 1.0; 95% CI 0.57–11.76; *p* = 0.99), Occurrence of myocardial infarction was significantly less frequent in TXA users than in nonusers (OR: 0.18; 95% CI 0.04–0.82; *p* = 0.03).

**Conclusion:**

TXA can effectively improve the recurrence rate of CDSH. It provides a high level of evidence-based medicine for clinical treatment. In addition, multicenter randomized controlled trials, with dose adjustments, are still needed to determine whether TXA intervention improves neurological function or prognosis.

## Introduction

Chronic subdural hematoma (CSDH) is frequently a result of head trauma, particularly among the elderly. Studies report an incidence rate as high as 20.6 per 100,000 individuals annually (1), With the aging global population, a marked increase in CSDH cases is anticipated (2). The enlarged subdural space, a consequence of cerebral atrophy common in the elderly, often coexists with the use of oral anticoagulants in this demographic (3). Head trauma typically leads to the rupture and subsequent bleeding of bridging veins. An initial collection of liquefied hematoma develops in the subdural space, encapsulated by a geomembrane rich in capillaries after about three weeks. This membrane is susceptible to recurrent bleeding, which in turn compresses the brain tissue, manifesting as headache, dizziness, and a range of neurological symptoms (4–6).

Surgical intervention through trepanation significantly mitigates these symptoms; however, CSDH frequently recurrence (33%) and is associated with a grim prognosis (7). The mechanisms underlying reoccurrence remain enigmatic, though prevailing theories implicate inflammation (8, 9), angiogenesis (10, 11), and hyperfibrinolysis (12, 13). Therapeutics developed following these theories, including statins and glucocorticoids, have proven effective at reducing postoperative recurrence by targeting inflammation and angiogenesis (14, 15). Nonetheless, recurrence rates remain disconcertingly high, necessitating the discovery of novel treatment targets.

Hyperfibrinolysis leads to the excessive breakdown and liquefaction of blood clots, impeding their reabsorption (16). Tranexamic acid (TXA), an antifibrinolytic agent, is postulated to inhibit the rapid dissolution of blood clots, thereby potentially preventing the recurrence of chronic subdural hematoma (CSDH) and reducing the need for multiple surgical interventions (17). Currently, the therapeutic efficacy of TXA following CSDH surgery is under investigation by numerous researchers through clinical trials. However, the outcomes are still subject to debate, (18–20), pointing to a pressing requirement for rigorous evidence-based medical research. In this context, our study synthesizes the existing data to assess whether TXA administration effectively curtails the recurrence rates of CSDH in affected patients.

## Materials and methods

### Systematic review

Systematic reviews are conducted by searching PubMed, Embase, Cochrane libraries, and CNKI providing broad access to literature, regardless of year or language. The Medical Subject Headings (Meshi) and the search terms were combined with Boolean logical operators using “Chronic subdural hematoma,” “Tranexamic acid,” “Prospective cohort studies,” “Randomized controlled trials,” “Retrospective cohort studies,” and other relevant synonyms.

### Selection criteria

All eligible citations were evaluated, and citations that did not meet the inclusion criteria or were repeatedly included were excluded. Read the full text carefully to further evaluate the relevance of the article. In addition, the references in the included articles are evaluated for further exploration of relevant research. All references In Endnote X9 (Research Soft, Philadelphia, United States).

### Inclusion and exclusion criteria

The inclusion criteria were as follows: (1) All enrolled patients were diagnosed with Chronic Subdural Hematoma; (2) Comparative studies include randomized controlled trials or prospective studies; (3) At least 16 patients were included in each trial; (4) report key outcome indicators. The exclusion criteria were as follows: (1) recurrent Chronic Subdural Hematoma (2) age under 18 years. The primary outcome measure was the recurrence rate in patients with Chronic Subdural Hematoma. We considered mortality, thrombosis and myocardial infarction as secondary outcomes. Recurrence was defined as the onset of symptomatic Chronic Subdural Hematoma during the study period, requiring a new intervention (based on radiologically and through clinical assessment).

### Data extraction and quality assessment

Two authors (Wani Pan and Jinyang Hu) independently extracted and Summarized data eligible for inclusion and exclusion Standards. Analyze demographic characteristics and data from all included articles. Relevant data such as study name, author, year of publication, country, region, and basic characteristics were extracted as baseline data.

Study quality was assessed using the software Review Manager (Version 5.4), which is a tool for evaluating the risk of bias in the included studies.

### Statistical analyses

Revan v5.4. was used to assess the overall recurrence rate. A random-effects model was used to assess pooled ORs, with the Mantel–Haenszel estimation method applied. Cochran Q (Chi-square) test and I^2^ statistics were used to assess inter-study heterogeneity. The heterogeneity was considered to be moderate if *I*^2^ values were > 25%. significance was determined using 95% CIs or *p* < 0.05 (22).

## Results

### Study identification and patient characteristics

**Figure 1:**
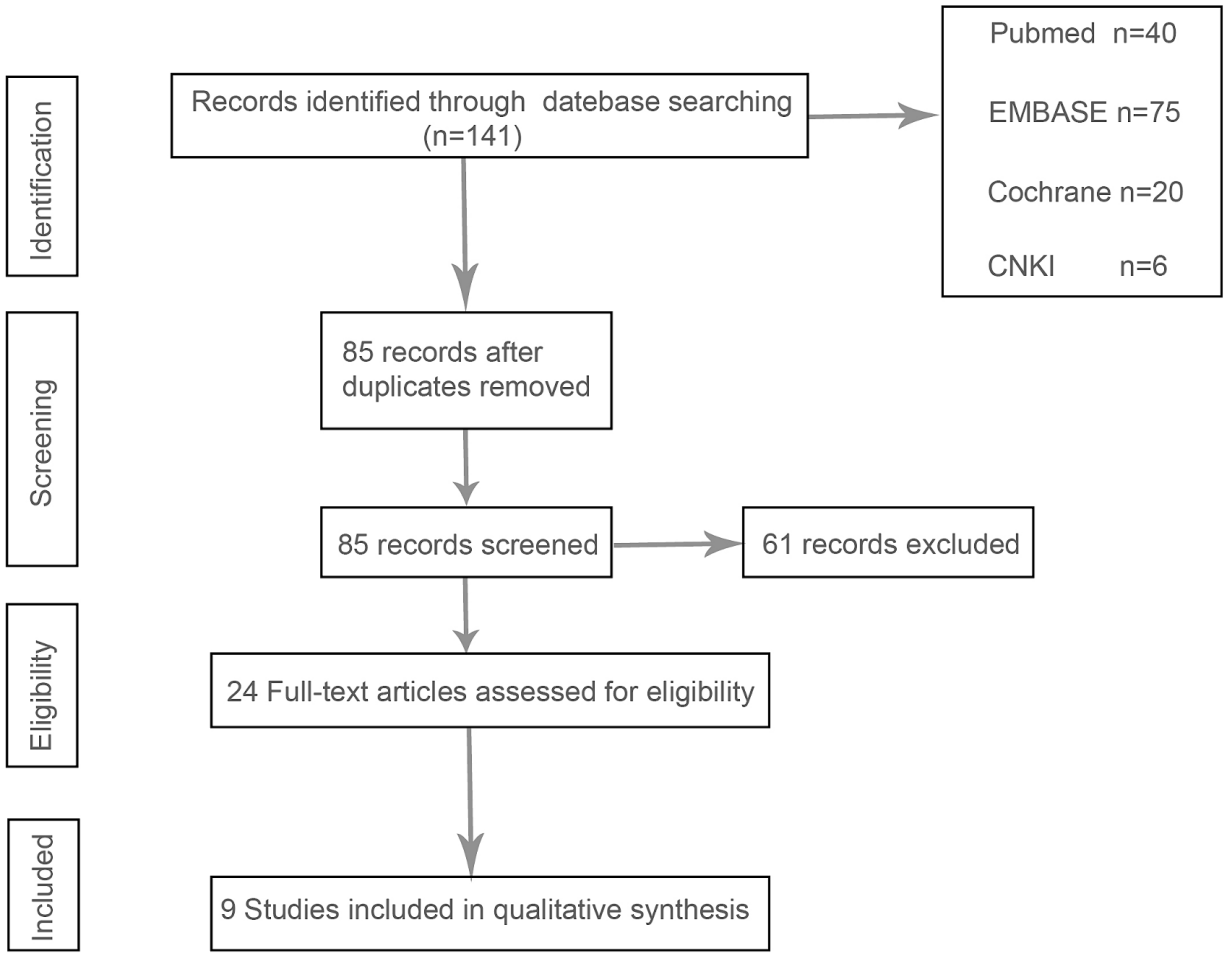
PRISMA flow chart.

After a systematic review of the literature, 141 literatures were initially screened, and after further screening, 9 literatures were finally included. Figure 1 shows the process of document selection. The included studies were published between 2012 and 2023. Table 1 summarizes the main characteristics and pharmacological interventions of the participants in the 9 included trials. Patients in each study were patients with CSDH. Five articles were RCTs, and 3 articles were prospective studies,1 article was retrospective. Treatment time varied from 1 to 12 weeks, 7 articles used burr holes,1 article was treated by drilling or craniotomy, and the remaining 1 article was conservative treatment. We summarize the main data from the included trials in Table 2. The results showed that all trials reported recurrence rates, with an overall recurrence rate of approximately 12.3% (5.7–32%) in the intervention group and 6.4% (range 1.4–18%) in the control group.

**Table 1:** Characteristics of included studies.

Publication	Study design	Treatments and sample size	Mean age	Gende (male, %)	Basic treatment	Doses	Treatment duration	Recruiting area
Xie et al. (22)	Prospective	TXA = 25 versus SNT = 25	40–81 (60.4) versus 38–80 (61.6)	13 (26)	Burr hole	1,500 mg once daily	1 weeks	China
Wan et al. (23)	RCT	TXA = 41 versus SNT = 49	72.02 ± 11.79 versus 69.57 ± 13.69	60 (66.7)	Burr hole or craniotomy	500 mg twice daily	3 weeks	Singapore
Yamada and Natori (24)	RCT	TXA =72 versus SNT = 82	78.2 ± 9.2 versus 78.8 ± 10.8	100 (64.9)	Burr hole	750 mg three times per day	12 weeks	Japan
Wakabayashi et al. (25)	RCT	TXA =50 versus SNT = 49	None	None	Burr hole	750 mg per day	4 weeks	Japan
Shibahashi et al. (26)	Prospective	TXA =6,564 versus SNT = 6,564	40–89 (75.6) versus 40–89 (75.5)	9,067 (69.1)	Burr hole	750 mg per day	Started oral TXA within 2 days after surgery	Japan
Miyakoshi et al. (27)	Retrospective	TXA =465 versus SNT = 465	81.3 (7.3) versus 81.1 (6.9)	606 (65.2)	Burr hole	750 mg per day	2 weeks	Japan
de Paula et al. (20)	RCT	TXA =24 versus SNT = 26	75.8 ± 11.8 versus 72.6 ± 11.9	31 (62)	Burr hole	750 mg three times per day	12 weeks	Germany
Yang et al. (28)	Prospective	TXA =41 versus SNT = 114	72 (65–83) versus 71.5 (60–79)	35 (77)	Burr hole	750 mg per day	7 weeks	Korea
Workewych et al. (29)	RCT	TXA =11 versus SNT = 13	70.18 (12.03) versus 70.85 (9.31)	70.9 (46)	Burr hole	500 mg three times per day	8 weeks	Canada

TXA, tranexamic acid, RCT, randomized controlled study, None, not reported. SNT, standard neurosurgical treatment.

**Table 2:** Recurrence rates included in the study.

Publication	Recurrence rates (%)		OR or HR (95%CI)	*p*-value
	Control	Intervention		
Xie et al. (22)	32	8	Not reported	<0.05
Wan et al. (23)	10.2	4.8	0.51 (0.11–2.47)	0.221
Yamada and Natori (24)	9.8	1.4	Not reported	0.083
Wakabayashi et al. (25)	5.7	10.9	Not reported	<0.05
Shibahashi et al. (26)	6.1	1.9	Not reported	<0.001
Miyakoshi et al. (27)	16.8	6.8	0.38 (0.26–0.56)	<0.05
de Paula et al. (20)	8.3	3.8	Not reported	0.5
Yang et al. (28)	7	2.4	Not reported	<0.05
Workewych et al. (29)	15	18	Not reported	1

### Risk of bias quality assessment

**Figure 2:**
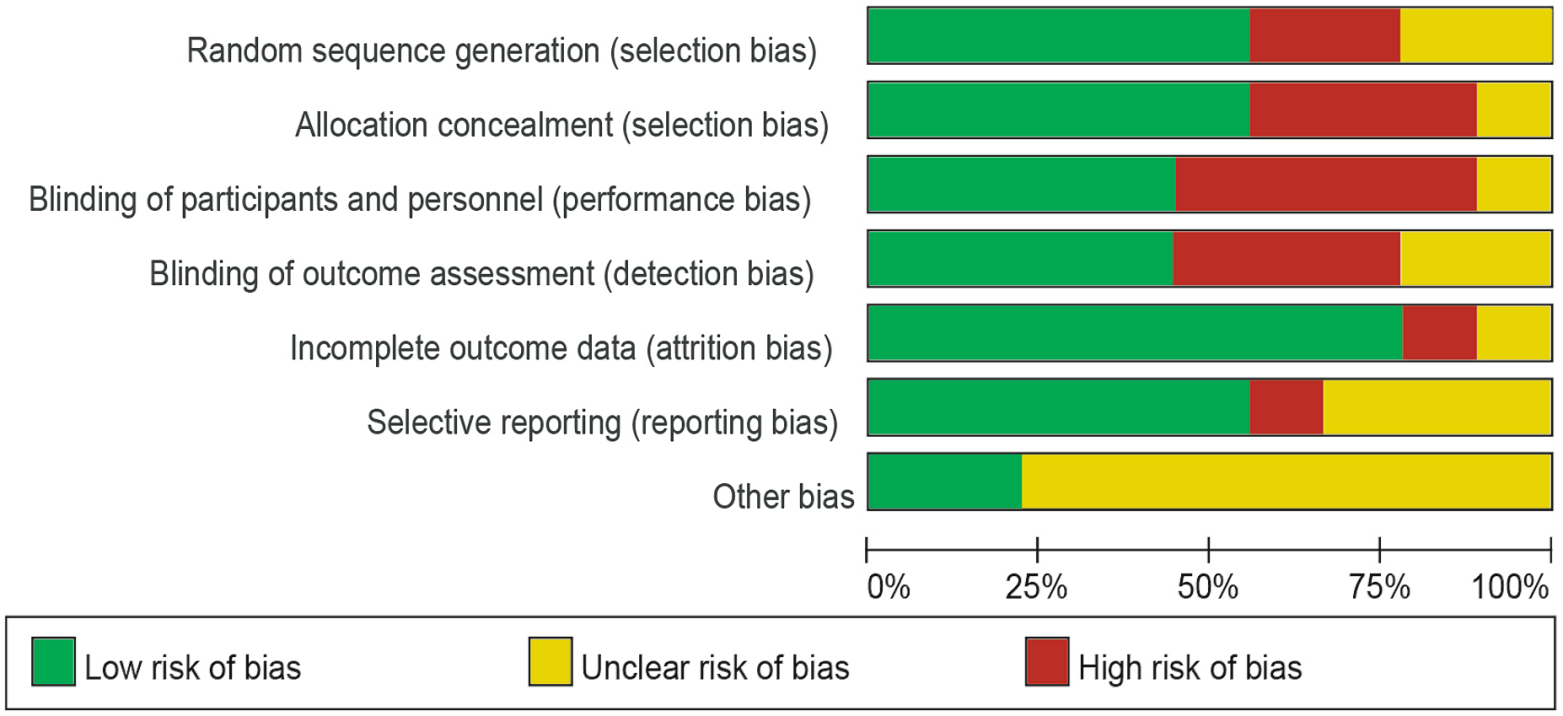
Risk of bias graph: the judgments about each risk of bias item are presented as percentages across all included studies.

**Figure 3:**
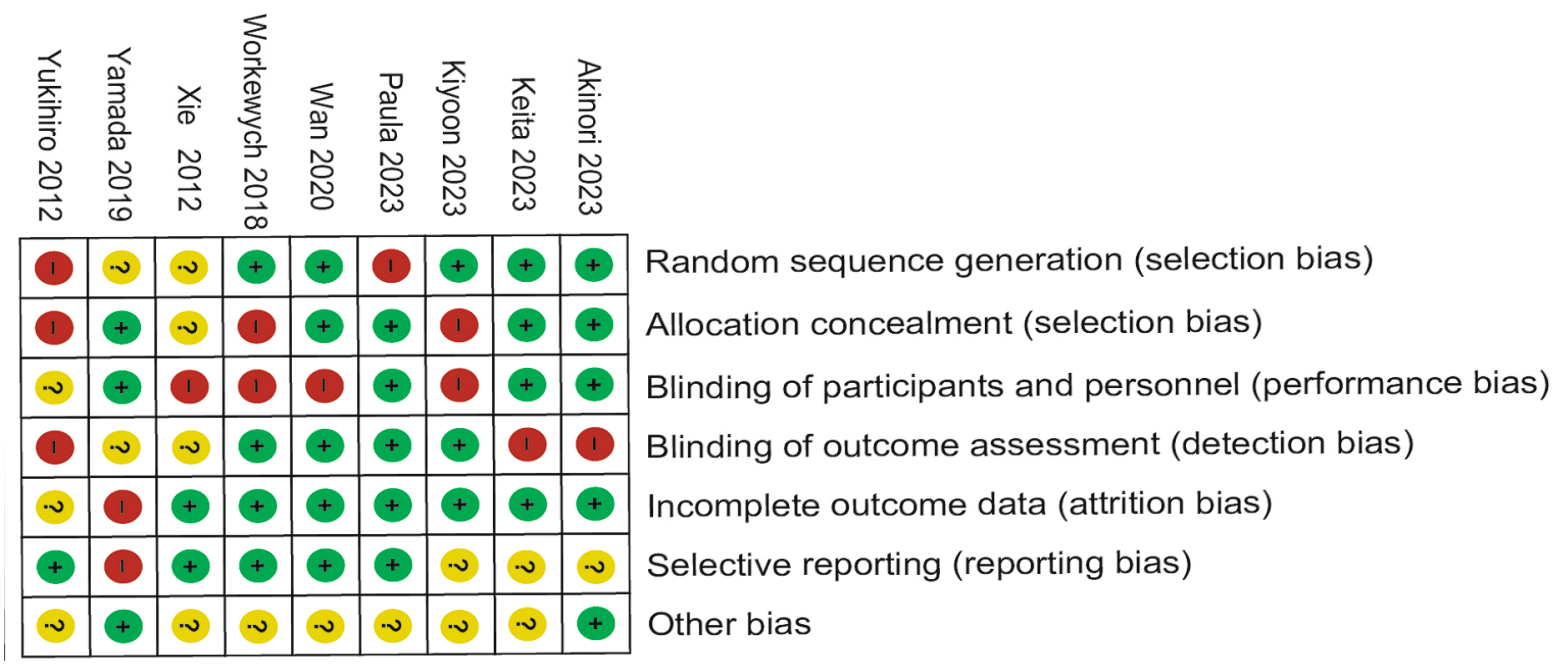
Risk of bias summary: the judgments about each risk of bias item for each included study.

Of the nine trials included, some trials were described in detail Random sequence generation with a low risk (23–29), Blinding of outcome assessment resulted in an unclear risk in some of the included studies, which may have led to detection bias (22). Some studies were scored high risk or unclear risk because of incomplete outcome data (24, 25). Individual bias and population bias at study level quality were, respectively, summarized in Figures 2, 3.

### The details of surgical treatment

Specific surgical protocols were not descripted in the included literature, Here, we describe in detail the procedure for trepanation and drainage of chronic subdural hematoma. First, select the appropriate anesthesia method after the clear indication of surgery. According to the preoperative imaging location, scalp and subcutaneous tissue were cut at the thickest part of the hematoma, the dura was cut after drilling, and the drainage tube was quickly inserted and then fixed by subcutaneous suture. Warm saline repeatedly flushes the hematoma cavity until the outflow liquid is basically clear. Finally, connect the drainage tube with an external drainage bag (30).

### Meta-analysis for recurrence rate and secondary outcomes

**Figure 4:**
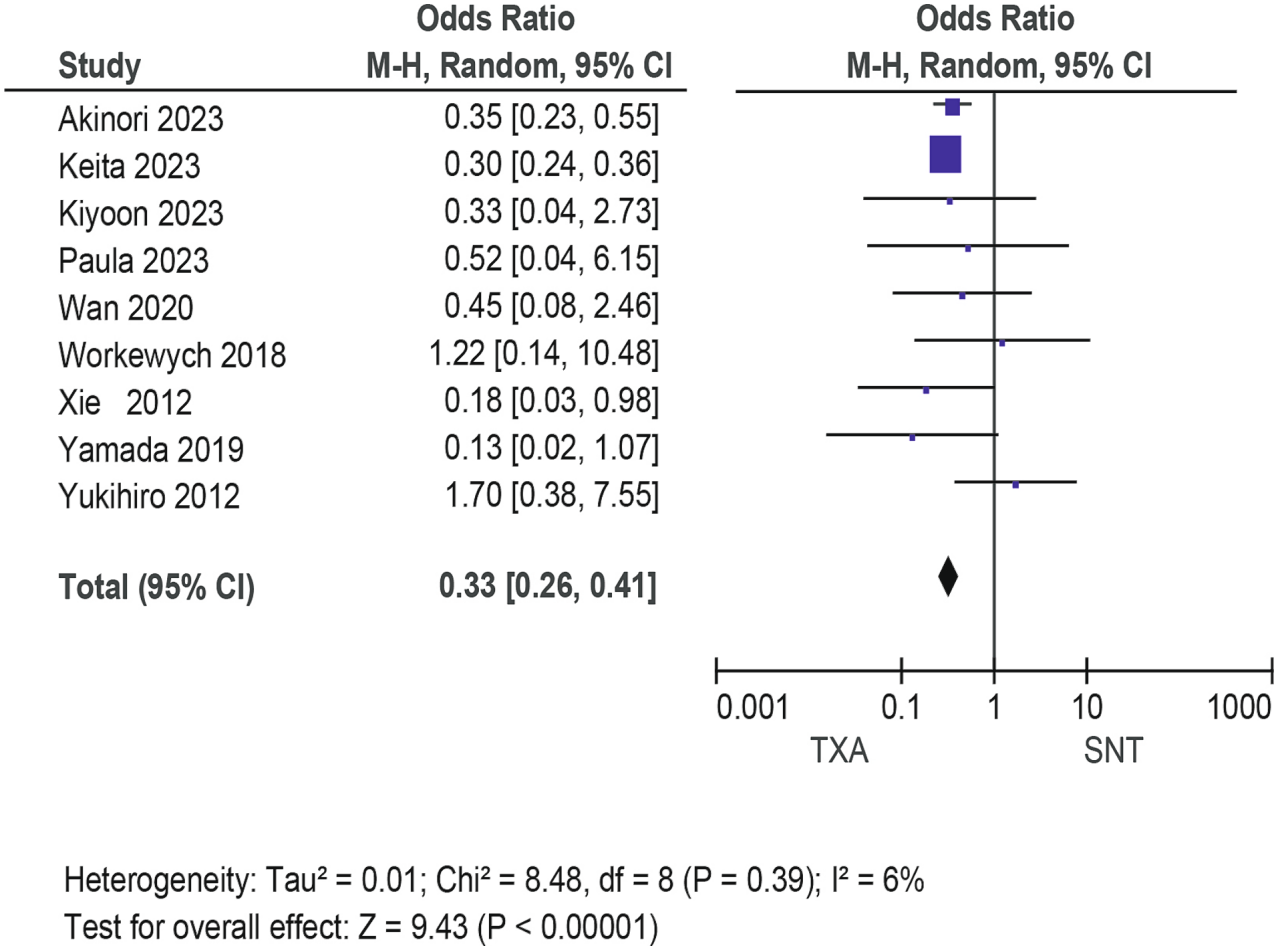
The efficacy of the experimental group was compared with that of the control group.

**Figure 5:**
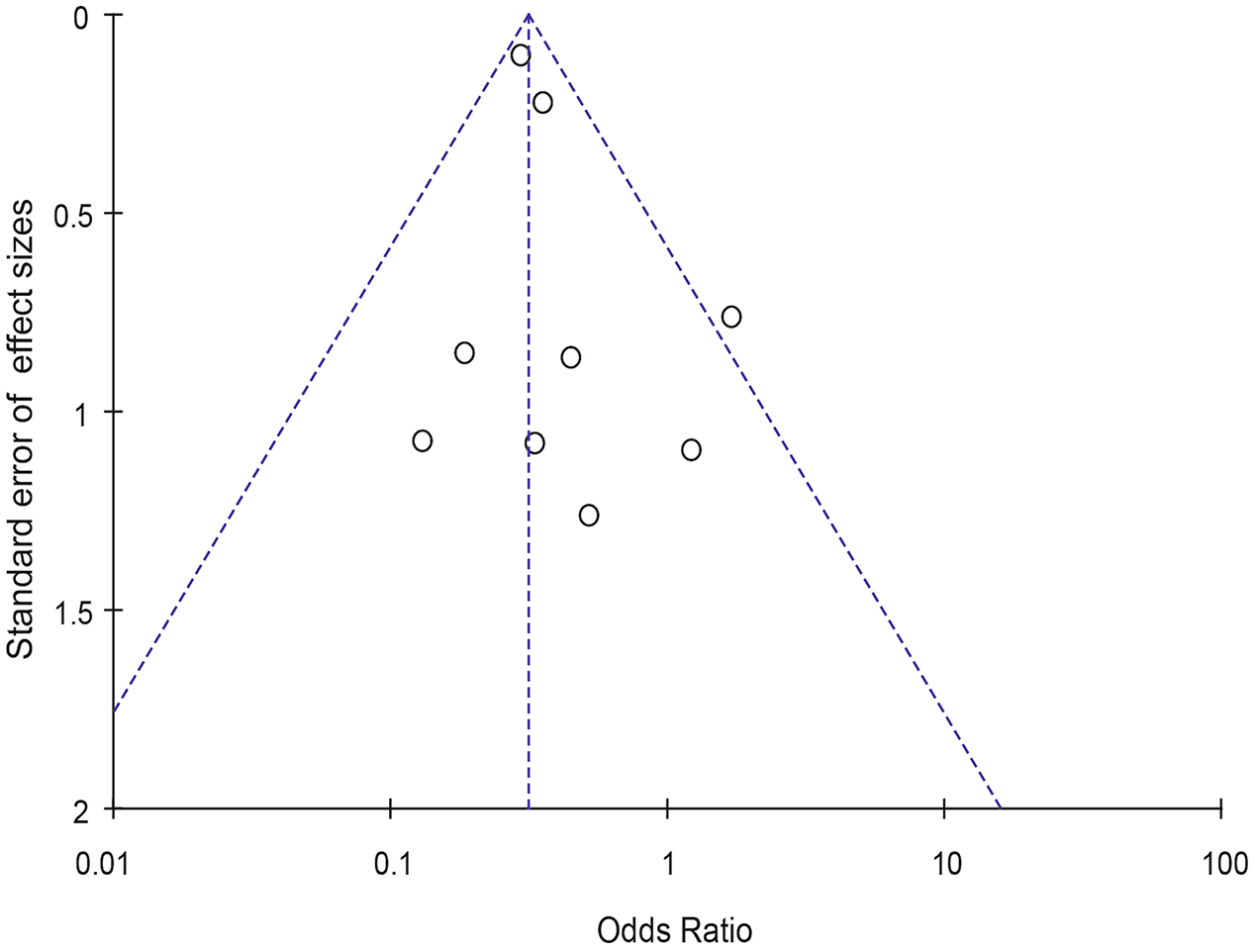
Funnel plot for detecting and displaying system heterogeneity.

Meta-analysis of patients with newly diagnosed Chronic subdural hematoma revealed a significantly improved recurrence rate when patients were treated with Tranexamic acid compared with standard neurosurgical treatment alone (OR: 0.33; 95% CI 0.26–0.41; *p* < 0.00001) Heterogeneity among studies was low (*I*^2^ = 6%, *p* = 0.39; Figure 4). There was no significant difference in the incidence rates of thrombosis (OR: 0.84; 95% CI 0.63–1.12; *p* = 0.23) and mortality (OR: 1.0; 95% CI 0.57–11.76; *p* = 0.99), Occurrence of myocardial infarction was significantly less frequent in TXA users than in nonusers (OR: 0.18; 95% CI 0.04–0.82; *p* = 0.03; Table 3). The funnel plot shows that there are some asymmetrical scattering points in the inverted funnel plot, which indicates that there may be some publication bias (Figure 5).

**Table 3:** Comparison of mortality, thrombosis, and myocardial infarction in the tranexamic acid group vs. the control group.

A						
Publication	Thrombosis				Weight	Risk radio (95%CI)
	TAX		Control			
	Events	Total	Events	Total		
Shibahashi et al. (26)	7	6,564	6	6,564	7.00%	1.17 [0.39, 3.47]
Miyakoshi et al. (27)	63	465	79	465	92.40%	0.80 [0.59, 1.08]
de Paula et al. (20)	1	24	0	26	0.60%	3.24 [0.14, 75.91]
Total	71	7,053	85	7,055	100%	0.84 [0.63, 1.12]
Test for overall effect: *Z* = 1.19 (*p* = 0.23).						

B						
Publication	Mortality				Weight	Risk radio (95%CI)
	TAX		Control	
	Events	Total	Events	Total
Miyakoshi et al. (27)	24	465	25	465	98.10%	0.96 [0.54, 1.70]
de Paula et al. (20)	1	24	0	26	1.90%	3.38 [0.13, 87.11]
Total	25	489	25	491	100%	1.00 [0.57, 1.76]
Test for overall effect: *Z* = 0.01 (*p* = 0.99).						

C						
Publication	Myocardial infarction				Weight	Risk radio (95%CI)
	TAX		control			
	Events	Total	Events	Total		
Shibahashi et al. (26)	2	6,564	11	6,564	100%	0.18 [0.04, 0.82]
de Paula et al. (20)	0	24	0	26	–	–
Total	2	6,588	11	6,590	100%	0.18 [0.04, 0.82]
Test for overall effect: *Z* = 2.22 (*p* = 0.03).						

## Discussion

As the global population ages and the use of anticoagulants becomes more prevalent, the incidence and societal impact of CSDH are anticipated to rise, presenting an escalating public health concern (31). The definitive management of chronic subdural hematoma is a topic of ongoing discourse. Surgical intervention remains the sole established treatment option, yet it is associated with substantial recurrence and mortality rates, estimated at 10%, particularly in the elderly and frail demographic (32, 33), The underlying mechanisms contributing to these postoperative outcomes remain elusive.

Several studies have shown that inflammatory factors and chemokines (IL-6, IL-8, IL-10, MCP1, and TNF-α) are mediators of CSDH development and play a crucial role in hematoma enlargement (34, 35). Additional findings suggest high VEGF levels also increase micro angiogenesis and enhance vascular permeability (10). In addition, in the hematoma of patients with CSDH, the activation of plasmin’s leads to a significant increase in thrombosis regulatory protein, and then forms a state of high fibrinolysis, which promotes blood vessel leakage leading to promote CSDH progression (36). Based on the understanding of inflammation, angiogenesis, and hyperfibrinolysis in the development of CSDH, Several related studies have investigated the role of various medical adjuncts, such as atorvastatin (37, 38), dexamethasone (15, 39, 40), TXA (4, 12, 14, 18, 20, 40–43), etc. in reducing their postoperative recurrence rate. However, to date, there is no established best adjuvant treatment.

Currently, low doses of atorvastatin have been used by many neurosurgeons to promote CSDH absorption and improve prognosis and neurological recovery (38). Compared with atorvastatin, dexamethasone can improve recurrence better (14). Some scholars have found that dexamethasone treatment is associated with a lower recurrence rate of CSDH, but no effect of dexamethasone on improving neurological prognosis and reducing mortality has been observed (43). In addition, dexamethasone increased the risk of all-cause death from CSDH (relative risk = 1.96), and adverse events with dexamethasone are generally severe even when given at megadose (44). Recent studies have shown that TXA is less effective than dexamethasone, but more effective than atorvastatin (14). This offers great potential for TAX to treat CDSH as an adjuvant or combination therapy. Importantly, TXA has favorable security. The most common side effects were mild gastrointestinal symptoms and headache (4), and TXA may promote the formation of thrombosis, then the risk of vascular embolism, however, previous trials have shown that this adverse effect is not clinically significant at doses of 1-2 g, which was higher than the dose regimen used in our trials (45). Besides, drug–drug interactions rarely occur in TXA (42), providing a better potential option for older.

To the authors’ knowledge, no conventional meta-analysis has evaluated the efficacy of TXA in reducing CSDH recurrence. This is the first meta-analysis that investigates the role of TXA in reducing the recurrence rate of CSDH. Our results suggest that TXA can significantly reduce the recurrence rate of CDSH (OR: 0.33; 95% CI 0.26–0.41; *p* < 0.00001), and improve the clinical prognosis of patients. However, there are some drawbacks to our study. First, there are not enough randomized controlled trials or prospective studies of TXA interventions, so the evidence based on their efficacy is limited. Second, we did not analyze the side effects of TXA, which could affect clinical treatment strategies. Finally, the low quality of some trials may potentially threaten the validity of our analysis. In the future, multicenter randomized controlled trials are still needed to evaluate TXA as a single or combination intervention to improve neurological function or prognosis.

## Conclusion

In summary, our results show that TXA can effectively improve the recurrence rate of CDSH. It provides a high level of evidence-based medicine for clinical treatment. In addition, multicenter randomized controlled trials, with dose adjustments, are still needed to determine whether TXA intervention improves neurological function or prognosis.

## Data availability statement

The original contributions presented in the study are included in the article/supplementary material, further inquiries can be directed to the corresponding author.

## Author contributions

WP: Writing – original draft. JyH: Writing – review & editing. XH: Writing – review & editing, Data curation. EJ: Writing – review & editing, Data curation. LY: Writing – review & editing. JgH: Writing – review & editing. TL: Writing – review & editing, Project administration, Formal analysis, Data curation.

## References

[1] Yang W Huang J. Chronic subdural hematoma: epidemiology and natural history. Neurosurgeon Clin N Am. (2017) 28:205–10. doi: 10.1016/j.nec.2016.11.002 28325454

[2] Balser D Farooq S Mehmood T Reyes M Samadani U. Actual and projected incidence rates for chronic subdural hematomas in United States veterans administration and civilian populations. J neurosurgeon. (2015) 123:1209–15. doi: 10.3171/2014.9.JNS141550, PMID: 25794342 PMC4575892

[3] Fernandes de Oliveira M. Chronic subdural hematomas and pursuit of nonsurgical treatment alternatives. World neurosurgeons. (2019) 126:481–3. doi: 10.1016/j.wneu.2019.03.151, PMID: 30922902

[4] Takahashi K Ohbe H Yasunaga H. Adjuvant oral tranexamic acid and reoperation after burr hole surgery in patients with chronic subdural hematoma: propensity score-matched analysis using a nationwide inpatient database. J Neurosurg. (2023) 138:430–6. doi: 10.3171/2022.5.JNS22664, PMID: 35901677

[5] Huang YW Li ZP Yin XS. Intraoperative irrigation of artificial cerebrospinal fluid and temperature of irrigation fluid for chronic subdural hematoma: a systematic review and meta-analysis. Front Neurol. (2023) 14:1218334. doi: 10.3389/fneur.2023.1218334, PMID: 37483449 PMC10359978

[6] Tonetti DA Thomas AJ Bulsara KR. Middle meningeal artery embolization for chronic subdural hematoma: a review. Operat Neurosurg. (2023) 24:469–75. doi: 10.1227/ons.0000000000000656 36897095

[7] Zhang J. Expert consensus on drug treatment of chronic subdural hematoma. Chin Neurosurg J. (2021) 7:47. doi: 10.1186/s41016-021-00263-z, PMID: 34809712 PMC8607705

[8] Hong HJ Kim YJ Yi HJ Ko Y Oh SJ Kim JM. Role of angiogenic growth factors and inflammatory cytokine on recurrence of chronic subdural hematoma. Surg Neurol. (2009) 71:161-5. doi: 10.1016/j.surneu.2008.01.023 18423527

[9] Frati A Salvati M Mainiero F Ippoliti F Rocchi G Raco A . Inflammation markers and risk factors for recurrence in 35 patients with a posttraumatic chronic subdural hematoma: a prospective study. J Neurosurg. (2004) 100:24–32. doi: 10.3171/jns.2004.100.1.0024, PMID: 14743908

[10] Hohenstein A Erber R Schilling L Weigel R. Increased mRNA expression of VEGF within the hematoma and imbalance of angiopoietin-1 and -2 mRNA within the neomembranes of chronic subdural hematoma. J Neurotrauma. (2005) 22:518–28. doi: 10.1089/neu.2005.22.518, PMID: 15892598

[11] Osuka K Ohmichi Y Ohmichi M Honma S Suzuki C . Angiogenesis in the outer membrane of chronic subdural hematomas through thrombin-cleaved Osteopontin and the integrin α9 and integrin β1 signaling pathways. Biomedicines. (2023) 11:1440. doi: 10.3390/biomedicines11051440, PMID: 37239111 PMC10216439

[12] Tanweer O Frisoli FA Bravate C Harrison G Pacione D Kondziolka D . Tranexamic acid for treatment of residual subdural hematoma after bedside twist-Drill evacuation. World Neurosurg. (2016) 91:29–33. doi: 10.1016/j.wneu.2016.03.062, PMID: 27032521

[13] Yoshikawa K Fujisawa H Kajiwara K Fujii M Kato S . Cause of hematic cysts of the orbit: increased fibrinolysis and immunohistologic expression of tissue plasminogen activator. Ophthalmology. (2009) 116:130–4. doi: 10.1016/j.ophtha.2008.08.041, PMID: 19019445

[14] Yu W Chen W Jiang Y Ma M Zhang W Zhang X . Effectiveness comparisons of drug therapy on chronic subdural hematoma recurrence: a Bayesian network Meta-analysis and systematic review. Front Pharmacol. (2022) 13:845386. doi: 10.3389/fphar.2022.845386, PMID: 35401183 PMC8993499

[15] Hutchinson PJ Edlmann E Bulters D Zolnourian A Holton P Suttner N . Trial of dexamethasone for chronic subdural hematoma. N Engl J Med. (2020) 383:2616–27. doi: 10.1056/NEJMoa2020473, PMID: 33326713

[16] Holl DC Volovici V Dirven CMF Peul WC van Kooten F Jellema K . Pathophysiology and nonsurgical treatment of chronic subdural hematoma: from past to present to future. World Neurosurg. (2018) 116:402–411.e2. doi: 10.1016/j.wneu.2018.05.037, PMID: 29772364

[17] de Faria JL da Silva Brito J Costa ESLT Kilesse C de Souza NB Pereira CU . Tranexamic acid in neurosurgery: a controversy indication-review. Neurosurg Rev. (2021) 44:1287–98. doi: 10.1007/s10143-020-01324-0, PMID: 32556832

[18] Miyakoshi A Nakatani E Kaneda H Hawke P Sasaki H Urano T . Administration of Tranexamic Acid after Burr Hole Craniotomy Reduced Postoperative Recurrence of chronic subdural hematoma in a Japanese regional population. Neurosurgery. (2023) 93:1160–7. doi: 10.1227/neu.0000000000002558, PMID: 37288980

[19] Wakabayashi Y Yamashita M Asano T Yamada A Kenai H Kondoh Y . Effect of Gorei-san with tranexamic acid for preventing recurrence of chronic subdural hematoma. No shinkei geka Neurological surgery. (2012) 40:967–71. PMID: 23100384

[20] de Paula M Ribeiro BDC Melo MM de Freitas PVV Pahl FH de Oliveira MF . Effect of postoperative tranexamic acid on recurrence rate and complications in chronic subdural hematomas patients: preliminary results of a randomized controlled clinical trial. Neurosurg Rev. (2023) 46:90. doi: 10.1007/s10143-023-01991-9, PMID: 37071217 PMC10111300

[21] Higgins JP Thompson SG Deeks JJ Altman DG. Measuring inconsistency in meta-analyses. BMJ (Clinical research ed). (2003) 327:557–60. doi: 10.1136/bmj.327.7414.557 PMID: 12958120 PMC192859

[22] Xie Q Xiaojun FU Xinlong XU. Clinical study of tranexamic acid combined and burr hole drainage with irrigation treatments for chronic subdural hematoma. ZH J J Traumatic. (2012) 17. doi: 10.3969/j.issn.1009-7147.2012.05.004 PMID: 12958120

[23] Wan KR Qiu L Saffari SE Khong WXL Ong JCL See AA . An open label randomized trial to assess the efficacy of tranexamic acid in reducing post-operative recurrence of chronic subdural haemorrhage. J Clin Neurosci Off J Neurosurg Soc Aust. (2020) 82:147–54. doi: 10.1016/j.jocn.2020.10.053, PMID: 33317724

[24] Yamada T Natori Y. Prospective study on the efficacy of orally administered tranexamic acid and Goreisan for the prevention of recurrence after chronic subdural hematoma Burr hole surgery. World Neurosurg. (2020) 134:e549–53. doi: 10.1016/j.wneu.2019.10.134, PMID: 31678452

[25] Wakabayashi Y Yamashita M Asano T Yamada A Kenai H Kondoh Y . [Effect of Gorei-san with tranexamic acid for preventing recurrence of chronic subdural hematoma]. No Shinkei Geka. (2012) 40:967–71., PMID: 23100384

[26] Shibahashi K Ohbe H Yasunaga H. Adjuvant oral tranexamic acid and reoperation after burr hole surgery in patients with chronic subdural hematoma: propensity score-matched analysis using a nationwide inpatient database. J Neurosurg. (2022) 138:430–436., PMID: 35901677 10.3171/2022.5.JNS22664

[27] Miyakoshi A Nakatani E Kaneda H Hawke P Sasaki H Urano T . Administration of Tranexamic Acid After Burr Hole Craniotomy Reduced Postoperative Recurrence of Chronic Subdural Hematoma in a Japanese Regional Population. J Neurosurg. (2023) 93:1160–1137., PMID: 37288980 10.1227/neu.0000000000002558

[28] Yang K Kim KH Lee HJ Jeong EO Kwon HJ Kim SH . Role of Adjunctive Tranexamic Acid in Facilitating Resolution of Chronic Subdural Hematoma after Surgery. J Korean Neurosurg Soc. (2023) 66:446–455. doi: 10.3340/jkns.2022.0200, PMID: 36325752 PMC10323266

[29] Workewych A Callum J Saarela O Montanera W Cusimano MD. Tranexamic acid in the treatment of residual chronic subdural hematoma: a single-centre, randomized controlled trial (TRACE). J. Neurotrauma. (2018) 35:A244–A245. doi: 10.1089/neu.2018.29013, PMID: 28306417

[30] Hwang Y Choi S Kim YS Park JS Choi JH Jeun SS . Comparative analysis of safety and efficacy in subperiosteal versus subdural drainage after burr-hole trephination for chronic subdural hematoma. Clin Neurol Neurosurg. (2022) 212:107068. doi: 10.1016/j.clineuro.2021.107068, PMID: 34847484

[31] Kolias AG Chari A Santarius T Hutchinson PJ. Chronic subdural haematoma: modern management and emerging therapies. Nat Rev Neurol. (2014) 10:570–8. doi: 10.1038/nrneurol.2014.163, PMID: 25224156

[32] Brennan PM Kolias AG Joannides AJ Shapey J Marcus HJ Gregson BA . The management and outcome for patients with chronic subdural hematoma: a prospective, multicenter, observational cohort study in the United Kingdom. J Neurosurg. (2017) 127:732–739. doi: 10.3171/2016.8.JNS16134.test, PMID: 27834599

[33] Soleman J Lutz K Schaedelin S Kamenova M Guzman R Mariani L . Subperiosteal vs subdural drain after Burr-hole drainage of chronic subdural hematoma: a randomized clinical trial (cSDH-drain-trial). Neurosurgery. (2019) 85:E825–e834. doi: 10.1093/neuros/nyz095, PMID: 31194877

[34] Stanisic M Aasen AO Pripp AH Lindegaard KF Ramm-Pettersen J Lyngstadaas SP . Local and systemic pro-inflammatory and anti-inflammatory cytokine patterns in patients with chronic subdural hematoma: a prospective study. Inflamm Res Off J Euro Histam Res Soc. (2012) 61:845–52. doi: 10.1007/s00011-012-0476-0, PMID: 22527446

[35] Araújo FA Rocha MA Mendes JB Andrade SP. Atorvastatin inhibits inflammatory angiogenesis in mice through down regulation of VEGF, TNF-alpha and TGF-beta1. Biomed Pharmacother. (2010) 64:29–34. doi: 10.1016/j.biopha.2009.03.003, PMID: 19811885

[36] Katano H Kamiya K Mase M Tanikawa M Yamada K. Tissue plasminogen activator in chronic subdural hematomas as a predictor of recurrence. J Neurosurg. (2006) 104:79–84. doi: 10.3171/jns.2006.104.1.79, PMID: 16509150

[37] Tang R Shi J Li X Zou Y Wang L Chen Y . Effects of atorvastatin on surgical treatments of chronic subdural hematoma. World Neurosurg. (2018) 117:e425–9. doi: 10.1016/j.wneu.2018.06.047, PMID: 29920396

[38] He C Xia P Xu J Chen L Zhang Q. Evaluation of the efficacy of atorvastatin in the treatment for chronic subdural hematoma: a meta-analysis. Neurosurg Rev. (2021) 44:479–84. doi: 10.1007/s10143-019-01218-w, PMID: 31953781

[39] Edlmann E Thelin EP Caldwell K Turner C Whitfield P Bulters D . Dex-CSDH randomised, placebo-controlled trial of dexamethasone for chronic subdural haematoma: report of the internal pilot phase. Sci Rep. (2019) 9:5885. doi: 10.1038/s41598-019-42087-z, PMID: 30971773 PMC6458174

[40] Shrestha DB Budhathoki P Sedhai YR Jain S Karki P Jha P . Steroid in chronic subdural hematoma: an updated systematic review and Meta-analysis Post DEX-CSDH trial. World Neurosurg. (2022) 158:84–99. doi: 10.1016/j.wneu.2021.10.167, PMID: 34728401

[41] Yang K Kim KH Lee HJ Jeong EO Kwon HJ Kim SH. Role of adjunctive tranexamic acid in facilitating resolution of chronic subdural hematoma after surgery. J Kor Neurosurg Soc. (2023) 66:446–55., PMID: 36325752 10.3340/jkns.2022.0200 PMC10323266

[42] Lodewijkx R Immenga S van den Berg R Post R Westerink LG Nabuurs RJA . Tranexamic acid for chronic subdural hematoma. Br J Neurosurg. (2021) 35:564–9. doi: 10.1080/02688697.2021.1918328, PMID: 34334070

[43] Holl DC Volovici V Dirven CMF van Kooten F Miah IP Jellema K . Corticosteroid treatment compared with surgery in chronic subdural hematoma: a systematic review and meta-analysis. Acta Neurochir. (2019) 161:1231–42. doi: 10.1007/s00701-019-03881-w, PMID: 30972566

[44] Wang X Song J He Q You C. Pharmacological treatment in the Management of Chronic Subdural Hematoma. Front Aging Neurosci. (2021) 13:684501. doi: 10.3389/fnagi.2021.684501, PMID: 34276343 PMC8280518

[45] Effects of tranexamic acid on death. Disability, vascular occlusive events and other morbidities in patients with acute traumatic brain injury (CRASH-3): a randomised, placebo-controlled trial. Lancet (London, England). (2019) 394:1713–23. doi: 10.1016/S0140-6736(19)32233-0 31623894 PMC6853170

